# Estimating Patient and Family Costs and CO2 Emissions for Telehealth and In-Person Health Care Appointments in British Columbia, Canada: Geospatial Mixed Methods Study

**DOI:** 10.2196/56766

**Published:** 2025-02-19

**Authors:** Graham Mainer-Pearson, Kurtis Stewart, Kim Williams, John Pawlovich, Scott Graham, Linda Riches, Sonya Cressman, Kendall Ho

**Affiliations:** 1 Digital Emergency Medicine Department of Emergency Medicine University of British Columbia Vancouver, BC Canada; 2 Rural Coordination Centre of British Columbia Vancouver, BC Canada; 3 Carrier Sekani Family Services West Vancouver, BC Canada; 4 Department of Family Practice University of British Columbia Vancouver, BC Canada; 5 First Nations Health Authority Vancouver, BC Canada; 6 School of Community and Regional Planning University of British Columbia Vancouver, BC Canada; 7 Patient Partner Researcher University of British Columbia Vancouver, BC Canada

**Keywords:** virtual care, economic evaluation, patient costs, lost productivity, informal caregiving, out-of-pocket costs, environmental costs, geospatial, patient, family, CO2, emission costs, health care, Canada, virtual service, emergency department, hospitalization, physician visit, consultation, sensitivity analysis, patient-paid, telehealth

## Abstract

**Background:**

Patients inevitably incur some cost for accessing health care, even in universal systems such as Canada. The COVID-19 pandemic dramatically shifted health care delivery from in-person to telehealth services, also shifting the proportion of costs offset by patients and their families by reducing the need to travel to in-person appointments.

**Objective:**

This study aimed to develop a method for estimating the costs patients and their families incur and CO_2_ emissions attributed to travel needed for emergency department (ED) visits, hospitalizations, and physician appointments.

**Methods:**

We present a method to evaluate the costs associated with in-person and telehealth care appointments from the perspective of patients, their families, and the environment. We used ED locations, road distances, and duration of appointment to account for costs paid by patients (ie, lost productivity, informal caregiving, and out-of-pocket expenses) attributed to travel to receive medical care. Costs to the environment were evaluated by calculating the amount of CO_2_ emitted per medical visit. Using our costs calculated per visit, we apply our method to calculate total patient costs for a simulated population over 1 year.

**Results:**

Our method estimates that patients in British Columbia pay up to $300 (2023 CAD, CAD $1=US $0.86) on average to attend an in-person ED visit, depending on where they live; $166 may be attributed to lost productivity, $83 to informal caregiving, and $50 to out-of-pocket expenses. These estimates are higher than most observed cost estimates. In addition, avoiding in-person care diverts up to 13 kg of CO_2_ per medical visit, depending on the distance and frequency of travel to appointments. This translates to up to $0.70 in carbon costs per visit, or cumulatively $44,120 per year in British Columbia, conventionally not included in patient cost estimates.

**Conclusions:**

We present a novel method for estimating patient-incurred costs and CO_2_ emissions from accessing health care and apply it to estimate that every year, patients in British Columbia pay upwards of 30 million dollars to access health care services, primarily for medical travel. Our method adds to the economic evaluation literature by providing a more comprehensive and context-modifiable calculation of patient costs that will allow for more informed decision-making regarding health care services.

## Introduction

Patients and their families incur costs when accessing health care services, even in universal, publicly funded systems such as Canada. However, patient-paid costs are often overlooked or underestimated in evaluations of health services because they are difficult to quantify and often considered out-of-scope for health policy decisions [1,2]. All patients pay to access health services either directly through co-pay cost-sharing arrangements or in other ways, such as needing to take time off work, travel to health care facilities, or attend an appointment as a caregiver. Methods to calculate and analyze patient costs are therefore of interest to account for the degree of cost-sharing that is passed off to patients and to protect them against financial hardship associated with receiving basic medical care. There are concerns about equity in cost-sharing because certain components contributing to patient-paid costs (ie, travel time or visit duration) may vary according to geographic location or service type, resulting in patient-paid costs that depend on where patients live and what type of service they access. Intergenerational inequities may arise from environmental costs to be paid in years to come, including greenhouse gas (GHG) emissions, such as CO_2_, associated with the need to travel to receive in-person service visits.

Over the past decade, studies on the patient-paid portion of health care costs have focused primarily on out-of-pocket expenses, such as travel costs [3-7]. Occasionally, some studies also consider time costs by accounting for lost productivity (ie, foregone paid or unpaid work) [8-13] and GHG emissions [13-19]. Studies on time costs and lost productivity have found that the majority of these costs are attributable to the amount of time patients spend attending appointments, estimating that patients pay between $80-$125 (2023 CAD, CAD $1=US $0.86) for their time to attend in-person medical appointments in developed countries [9,10,13]. Of the studies that accounted for GHG emissions and environmental costs, a systematic review found that 0.70-372 kg of CO_2_-equivalent emissions were avoided by telemedicine appointments [16]. Many of these studies are based on assumed values for key parameters, such as cost per unit of travel distance, wage, fuel efficiency, and CO_2_ emissions, for different patient groups defined by age, sex, or type of service accessed. Costs are typically presented either by determining per-visit costs or analyzing costs over a longer time horizon.

Based on costing methods currently available, we see a significant gap in estimating patient-paid costs. Current methods calculate costs for a specific population subgroup, often including patient data that prevents cost results from being generalizable to other cases. Furthermore, more general studies of patient costs do not include all aspects of costs; instead, they focus on a specific type of cost, resulting in an incomplete picture of the full burden of costs to patients. We propose to construct a multidimensional method for a cost analysis to calculate patient costs and do so in a way that is able to be objectively and consistently applied to different service models, be they in-person or telehealth. In doing so, we present an open-source analytical tool that can be used to calculate these costs generally, using easy-to-obtain or public datasets, and without the need for specific patient-level administration data or costly patient-reported data. Incorporating a geospatial component allows our method to function for both rural and urban areas and can be applied by other health systems to capture patient costs specific to their region. The advantage of this approach is that it provides a standardized way to calculate these costs that will allow for increased opportunities for economic evaluations and comparisons across different service approaches, such as in-person, telehealth, or hybrid care. The aim of this paper, thus, is to present a transferrable method to comprehensively account for patient costs to access health care, inclusive of lost productivity, informal caregiving, out-of-pocket costs, and GHG emissions.

## Methods

### Overview

The geospatial method was developed to calculate the per-visit costs for health service use from the perspective of patients, their family caregivers, and future generations. The method is inclusive of costs attributed to the receipt of care that are paid at the time of health service use but are not covered by the health care system, in alignment with methods used by the World Health Organization to measure progress on universal health systems’ objective to provide financial protection from the high costs of illness [20]. We calculate costs attributed to attending emergency departments (EDs), being admitted to the hospital, and receiving in-person or telehealth physician services. We use publicly available data on distances, appointment durations, and average wage statistics to determine productivity losses for paid or unpaid work with the human capital method of valuation. All data and code used in the method are available in a companion repository on GitHub [21]. Costs to receive care are expressed as service-specific unit costs, comprised of the subunits of lost productivity, informal caregiving, and out-of-pocket expenses, disaggregated by age and region. GHG emissions are expressed as the volume of CO_2_-equivalent emissions. All costs are reported in 2023 CAD, approximately equal to US $0.86. Results were simulated for individuals in British Columbia based on the age, region, and service use patterns observed from the real British Columbia population.

### Geospatial Evaluation

The geospatial method accounts for geography specific to British Columbia, a province in Canada with approximately 16% rural and 84% urban population (population density ranging between 0.01/km^2^ to 18804.48/km^2^) [22]. The geographic regions are defined as community health service areas (CHSA) and 5 broader health authority (HA) geographies. At the time of analysis, there were 218 CHSAs defined in British Columbia [23]. Our geospatial method operates primarily at the CHSA level, with results aggregated and presented at the HA level. Travel distances were calculated for hospitalizations, physician visits, and ED visits by determining the geographic center of each CHSA and then calculating the real street distance from each CHSA center to the nearest emergency department, assumed to be a proxy for the nearest location to access care, using road network data obtained through the *OpenStreetMap* package (version 4.0) and calculating with R (version 4.2; R Core Team). Travel duration was also calculated with this method, using speed limits of the specific roads traveled on each trip. The calculated travel distance and duration for each CHSA were aggregated to the HA level using the population-weighted mean of all CHSAs within each HA.

In addition, we also analyzed unit costs for CHSAs using an urban and rural stratification. In the Government of British Columbia record of all health regions, each CHSA is given a classification, numbering 1 through 7, of which 4 and below are considered urban and 5 and above are considered rural [22]. We then aggregated data for all CHSAs considered urban or rural and computed a unit cost for each. This involved calculating travel distance and duration, using our geospatial method, for both urban and rural CHSAs.

### Patient and Family Costs

#### Overview

British Columbia has a publicly funded, single-payer health system, where the provincial government pays for core health services. In our model, costs not paid by the health care system were defined as patient and family costs and were attributed to the following health service types: ED visits, hospital admissions, and telehealth or in-person physician encounters. Costs were calculated for each service type by age group and health authority. For ED visits, costs were calculated separately for Canadian Triage Acuity Scale (CTAS) levels I-III and IV-V [24]. The CTAS is a method for grouping patients according to the severity of their condition upon presentation to an ED, with level I being the most severe and level V being the least severe, resulting in differentials in the amount of time patients spend at ED. We used age groupings of 0-14, 15-64, and ≥65 years, which were chosen to distinguish the working-age population to account for lost productivity and align with Statistics Canada's definitions of working and retirement ages [25,26]. Average hourly wage rate data taken from Statistics Canada begins at age 15, which is consistent with the Employment Standards Act of British Columbia, which allows light work for persons aged 15 without a permit [27,28]. Each calculated cost for each service type and category is comprised of the following subunits: lost productivity, informal caregiving costs, and out-of-pocket expenses. A goal of the method is to create a unit cost database containing all costs, organized by age group, service type, and region.

#### Lost Productivity

Lost productivity was calculated from the time costs attributed to any foregone paid or unpaid work required to attend appointments or clinic visits. Time costs include time spent on travel, which was obtained by our travel distance calculations, time spent waiting to receive health services, and appointment time (see Multimedia Appendix 1 for details). For physician appointments, ED visits, hospitalizations, and telehealth visits, the total time was multiplied by the average hourly wage rate for British Columbia for 2022. Lost productivity costs were calculated for all working-age patients (15-64 years), and productivity costs were zero for individuals aged 0-14 and ≥65 years. For hospitalizations, due to the increased length of stay, we capped time costs for lost productivity at 8 hours per 24-hour period.

#### Informal Caregiving

Informal caregiving was evaluated from the time required for caregivers to accompany patients for physician appointments, ED visits, or admissions. For physician, ED, and telehealth visits, we assumed that 50% of all patients aged 15 years and older attend appointments with a caregiver, and 100% of patients in the 0-14 age group attend with a caregiver. We assumed that the caregiver would have the same time commitment for travel time, wait time, and appointment duration as the patient. For hospitalizations for admitted patients, we assumed that 25% of all patients in the 15-64 and ≥65 years age groups and 75% of patients in the 0-14 years age group have a caregiver present for the duration of their visit. For hospital admissions, the caregiver costs were prorated to 8 hours per day to account for lost wages only within a regular workday. Given the lack of clear estimates available in the published literature to draw from, we derived estimates from several Canadian sources and updated them with input from clinical study team members who see patients in clinical practice in order to estimate caregiver attendance percentages [29,30].

#### Out-of-Pocket Costs

The out-of-pocket subunit includes costs incurred by patients that are a direct result of the appointment but not for the appointment itself. This includes costs such as, but not limited to, vehicle costs, gas, parking, or accommodation. For physician visits, ED visits, and hospital admissions, direct travel costs were calculated based on standard Canadian rates for a compact car (eg, Toyota Corolla) with 80% highway and 20% urban road driving, gas prices of approximately $2 per liter, and average annual mileage of 20,000 km [31]. For physician and ED visits, accommodation costs were assumed to be $0 for all visits as none of the travel distances would require an overnight stay. For hospitalizations, we assumed accommodation would be needed for the caregiver for 2 days if the total travel distance was greater than 50 km to account for extra travel and time necessary on the patient’s admission and discharge days. As a result, accommodation costs were only included for hospitalizations in Northern Health Authority and equaled $0 for all other unit costs. When applicable, the accommodation cost was determined by averaging hotel costs in the area using Google Travel [32]. This accommodation cost was then multiplied by the appropriate caregiver attendance percentage, depending on the age group (75% for age <15 years, 25% for age ≥15 years) to estimate the final caregiver accommodation cost. For ED visits, one meal, at a $15 value, was assumed to be needed for patients in CTAS I-V. For hospitalizations, 3 meals per day for 2 days (admission and discharge days), at $15 value, was assumed to be needed for caregivers. Meal costs were then also multiplied by the appropriate attendance coefficient (75% for age <15 years, 25% for age ≥15 years) to estimate the final caregiver meal costs. Parking costs were determined using rates from a city within the relevant health authority. Average parking rates from the chosen city’s core were used for physician visits, and hospital rates were used for ED visits and hospitalizations. Furthermore, for hospitalizations, parking was assumed to be required for the caregiver for 2 days to align with the assumption made for accommodation costs.

#### Telehealth Visit Costs

Telehealth visit costs were similarly comprised of lost productivity, informal caregiving, and out-of-pocket subunits. However, as many expenses for in-person visits, such as travel-related costs, are not applicable to telehealth visits, the content of the subunits differ. The lost productivity and informal caregiving costs included only appointment wait time and duration, given that travel time is zero. For out-of-pocket expenses, instead of meals, parking, or direct travel costs, the telehealth visit cost included data usage costs. Data usage costs were obtained using estimates of data used for an average telehealth encounter length and using per-unit data costs from the least expensive plans available in British Columbia. Telephone and internet were both considered, based on the current modality of using telehealth care, with 80% weight given to telephone data costs and 20% weight to internet data costs to determine the final data usage rate. Multimedia Appendix 1 shows the details of the full data usage cost calculation. We assumed as a baseline that everyone has access to a phone, so the cost of a device was considered to be zero, and electricity costs were negligible.

### Greenhouse Gas Emissions

In-person visits also carry an environmental cost in the form of carbon costs from travel. We included this in our calculations by inputting total travel distance into an emissions calculator [33], using a fuel efficiency rate of 7.06 L/100 km for a 2022 Toyota Corolla. We obtained CO_2_-equivalent emissions, in kilograms, per visit for each health authority, based on the distance traveled for a visit in that region.

### Sensitivity Analysis

As our method requires assumptions to be made for certain parameter values, we have conducted a sensitivity analysis to explore how the cost output changes based on changes to specific parameters. We conducted the sensitivity analysis on 4 different parameters: wage, travel distance, travel duration, and caregiver attendance percentage. We tested the wage assumption using British Columbia minimum wage ($16.75) [34], $20, $25, $30.54 (base case), and each $5 increment from $35 to $50 (all CAD). The caregiver attendance percentage was tested for 0%, 25%, 50% (base case), 75%, and 100% of appointments attended. Travel distance and duration were varied in 10% increments from 50%-150% of the base case average value. The overall street distance calculation method was also compared with the haversine (straight-line) distance from the center of each CHSA to the nearest in-person visit location. When varying one parameter value, we kept all other parameters constant and applied the method to compare the resulting unit cost output to our base case.

### Simulated Population

To illustrate a potential application of this method, we simulated costs and CO_2_ footprint for 100,000 individuals distributed across age groups and regions (health authority). We used a Canadian Institute for Health Information (CIHI) report on ED visits to estimate the total number of ED visits per person in British Columbia in one year [35]. We then used NACRS data from British Columbia in 2022 to determine what proportion of ED visits were assigned CTAS I-III and CTAS IV-V in order to apply the appropriate unit cost [35]. For physician visits, we used an international report that found an average of 2.7 provider visits per year in Canada [36]. For telehealth visits, we used a cross-sectional study of telehealth care usage in Ontario, which found an average rate of 48 visits per 1000 people per week, which we then converted to 2.5 visits per person per year [14]. We then distributed these visits between age groups and regions, according to the population distribution in British Columbia, and simulated the impact across British Columbia. Costs were applied to determine the estimated total patient cost by service type for a 1-year period. In addition, we calculated GHG emissions for the simulated population over the 1-year period using the total distance traveled across all ED and physician visits.

### Ethical Considerations

All datasets analyzed in this study are publicly available, and their secondary use does not require human consent; therefore, ethics approval was not needed for this study as per the University of British Columbia’s Behavioral Research Ethics Board guidelines [37].

## Results

### Geospatial Evaluation

**Figure 1:**
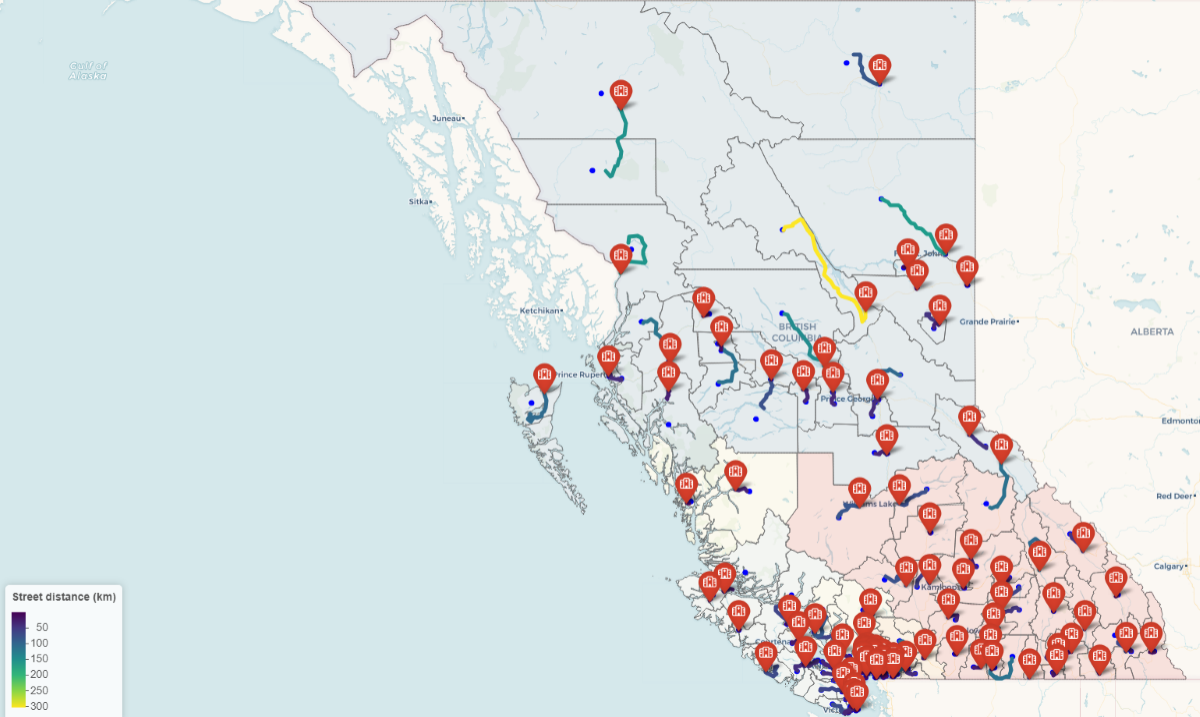
Map of calculated routes.

Travel distances and durations were calculated using our method for each CHSA in British Columbia. The longest distance calculated was 302.37 km for the Mackenzie CHSA, and the longest duration calculated was 429.58 minutes for the Mackenzie CHSA. The shortest distance was 0.59 km for the Fort Nelson Population Centre CHSA, and the shortest duration calculated was 1.21 minutes for the Fort Nelson Population Centre CHSA. Figure 1 shows the resulting map of all CHSA with their calculated route. The street distance calculation method uses a route beginning at the center of each CHSA. In cases where a street is not present, the method may deviate to find the nearest street. Distances and durations calculated from each CHSA were then aggregated to the HA level using a weighted mean based on the population of each CHSA, the results of which are presented in Table 1.

**Table 1:** Distance and duration by health authority.

Health authority	Weighted mean distance (km)	Weighted mean duration (min)	CO_2_-equivalent emissions per visit (kg)
Fraser	30.6	64	4.67
Interior	42.8	65.2	6.53
Northern	69.4	93.8	10.59
Vancouver Coastal	12.06	24.2	1.84
Vancouver Island	24.4	37.8	3.72

### Patient and Family Costs

Our method produced individual costs associated with each category (ie, each combination of age group, health authority, and service type), which we have defined as unit costs. Each unit cost, therefore, represents the total cost for one visit of that type. The cost database also includes the cost for each subunit: lost productivity, informal caregiving, and out-of-pocket costs (see Multimedia Appendix 2 for full database). The creation of the cost database allows for the comparison of unit costs across different categories. Costs can be compared between service types, regions, or age groups. From the cost database, we determined the range of costs across the different categories, from a minimum cost of $31 for a physician consult in Vancouver Coastal Health to a maximum cost of $300 for an ED visit in Northern Health.

**Figure 2:**
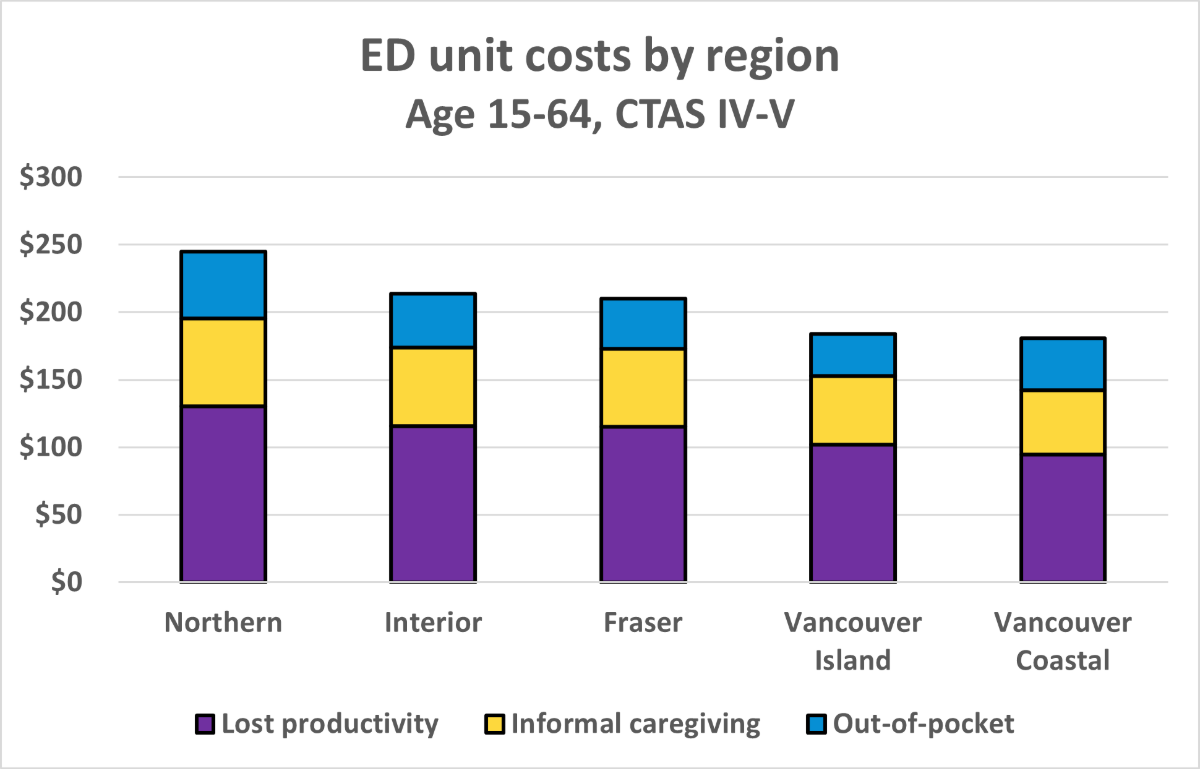
Single visit emergency department (ED) unit costs by region with subunit breakdown. CTAS: Canadian Triage Acuity Scale.

Visits to EDs with the highest urgency (CTAS IV-V) in the Northern Health region among patients in the 15-64 years age group had the highest costs per visit ($245), considerably higher than the amount for patients accessing the ED in regions covered by Vancouver Coastal Health ($181; Figure 2).

**Figure 3:**
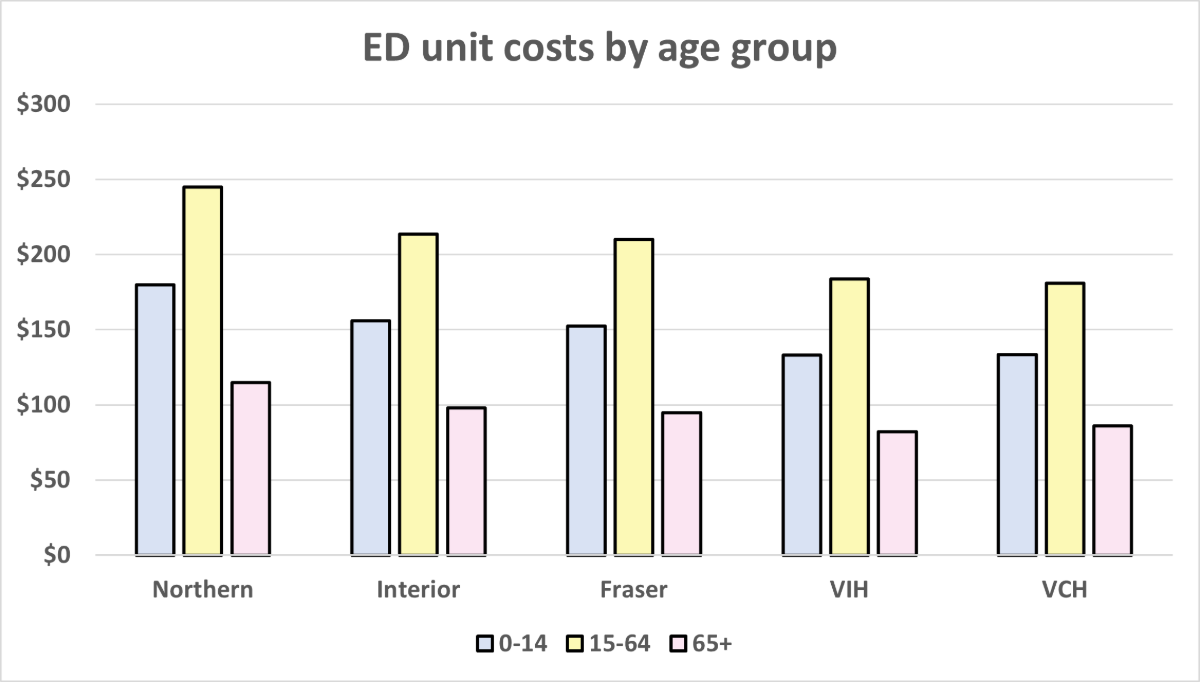
Single visit emergency department (ED) unit costs by age group. VIH: Vancouver Island; VCH: Vancouver Coastal.

**Figure 4:**
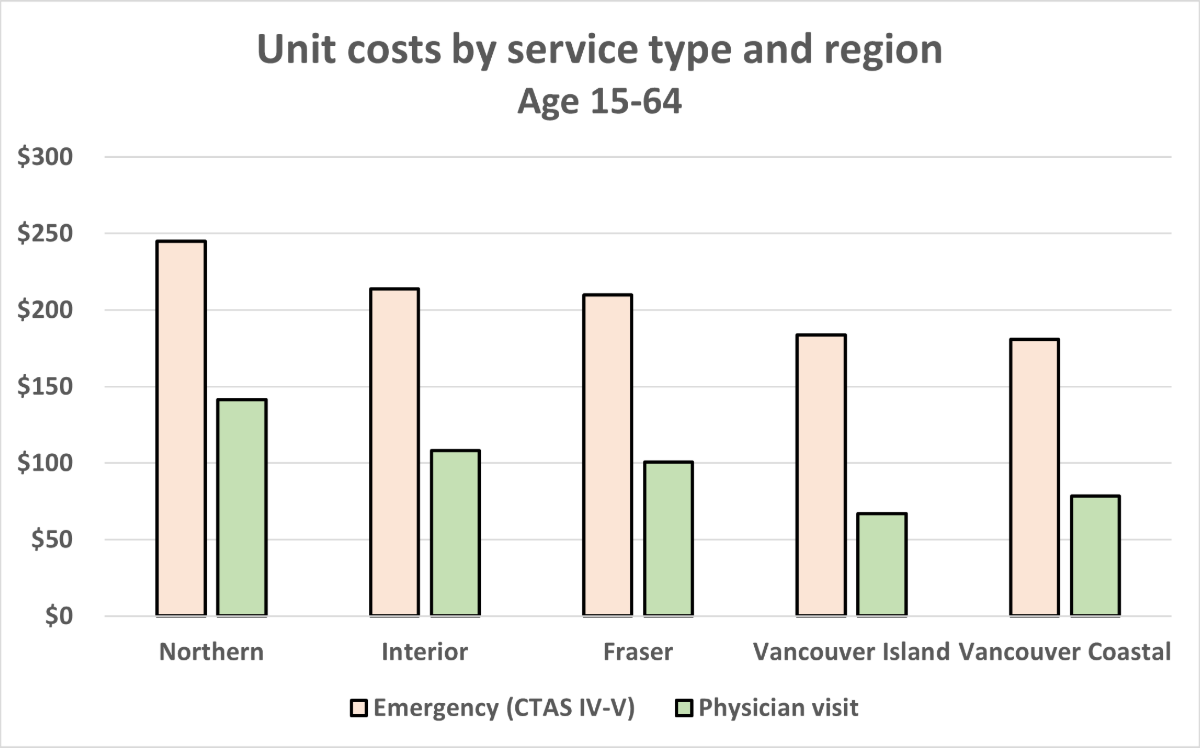
Single visit unit costs by service type and region. CTAS: Canadian Triage Acuity Scale.

Costs among the 15-64 years age group were driven by lost productivity. This finding relies on the assumption of negligible productivity impacts among the 0-14 and ≥65 years age groups and that the attendance of an informal caregiver is 100% for the 0-14 years group and 50% for the ≥65 years age group. However, when applied to a specific population, it is possible that absolute costs may differ, given that the population may be more heavily weighted toward one or more age groups. We also observe from Figures 3-4 that the geographic trend observed previously continues to hold, with the Northern Health region having the highest costs across all age groups and service types.

Comparing costs among patients in the age 15-64 group, ED visits at the CTAS IV-V level were higher across all health authorities, ranging from $181 to $245 compared with the estimated $67 to $142 estimated cost for patients to attend primary care appointments.

Unit costs for telehealth visits were found to be $30 for the 15-64 years age group. Given the lack of travel-related costs, telehealth visit unit costs did not vary geographically across the province.

In addition to the aggregation to the health authority level, we calculated unit costs based on a stratification of each CHSA between urban and rural, which are presented in Table 2. This involved aggregating 4 classes of urban CHSAs and 3 classes of rural CHSAs. We then calculated unit costs for physician visits and for ED visits in the CTAS I-III and CTAS IV-V categories. Given the longer travel distances and durations associated with rural locations, we expected to see that rural unit costs are higher than urban for all age groups for both ED and physician service types by a considerable margin.

**Table 2:** Base per-visit unit costs by urban and rural community health service area (CHSA) classification.

Urban						Rural					
Age group (years)	Physician visit (unit cost), $	Emergency medicine (unit cost), $			Age group (years)			Physician visit (unit cost), $		Emergency medicine (unit cost), $	
		CTAS^a^ I-III	CTAS IV-V						CTAS I-III		CTAS IV-V
0-14	52	157	117	0-14			146		250		211
15-64	72	222	162	15-64			196		345		286
≥65	32	92	72	≥65			96		156		136

^a^CTAS: Canadian Triage Acuity Scale.

### Greenhouse Gas Emissions

We also determined GHG emissions and costs associated with the travel distance for both the rural and urban CHSAs. We used the same reference vehicle and fuel efficiency (2022 Toyota Corolla, 7.06 L/100 km) and obtained kilograms of CO_2_-equivalent emissions per visit, which are shown in Table 3.

**Table 3:** Base per-visit emissions by urban and rural community health service area (CHSA) classification.

	Distance per visit (km)	CO_2_-equivalent emissions per visit (kg)
Rural	91.2	13.92
Urban	18.94	2.89

### Sensitivity Analysis

A sensitivity analysis was applied to show how the unit cost outputs change when parameter values are deterministically varied for each of the health services included. The magnitudes of the observed cost variations are similar relative to the base case for each service type and consistently represent a linear 2%-3% change in total unit cost for every $1 change in wage (see Multimedia Appendix 2). Costs in the Northern Health authority had the largest variation and represented the maximum of the range of changes observed. A noteworthy caveat is that although a lower wage value results in lower total costs, this may still represent a larger burden to patients with this income profile. In the case of minimum wage, visit costs are approximately 3.5-4 times the cost of 1 hour of work, whereas in our base case using British Columbia average wage, visit costs are approximately 3-3.5 times the cost of 1 hour of work.

We also varied assumptions about the percentage of appointments attended by a caregiver and found that each 25% change in caregiver attendance resulted in changes of less than $35 in total, except for hospitalizations; the method was sensitive to assumptions about how often a patient has a caregiver attend. The impact changed the estimated patient and family costs by $434-$494, indicating the importance both in the magnitude of this cost overall and also in the significant amount of time that caregivers devote to supporting patients in the hospital. Similar to the analysis of wage changes, costs in Northern Health authority had the largest variation based on a change in caregiver attendance percentage. The observed change in costs for each service type was consistent across all age groups in terms of absolute value. Given that the base case unit costs differ for each service type and age group, the observed change in costs represents a difference in relative change even when the absolute values remain constant. For the 0-14 years age group, a 25% variation in caregiver attendance percentage represents approximately a 17-32% variation in unit costs when compared to the base case. For the 15-64 age group, costs varied by approximately 12.5%-22% compared with the base case. Finally, for the ≥65 years age group, costs varied by 28%-94% compared with the base case. The ≥65 years age group shows significant variation due to 25% caregiver attendance representing a large variation in the absolute value of the unit cost, particularly for hospitalizations, combined with informal caregiving costs being a larger proportion of this age group’s total unit cost. Without considering the hospitalization service type, costs varied by approximately 28%-35% for the ≥65 years age group.

Our analysis of the method’s travel parameters found that a 10% variation in distance and duration resulted in a change in cost ranging from $2-$8 for patients aged 0-14, $2-$10 for patients aged 15-64 years, and $1-$6 for patients aged ≥65 years. The magnitude of variation remained consistent across all service types. Similar to other parameters included in the sensitivity analysis, the largest variation in costs was observed in the Northern Health authority. While the magnitude of cost variation was consistent for all service types, the relative change in costs did vary due to the difference in the base cost. The observed variation in costs from a 10% change in travel distance and duration represented a change of approximately 0.5%-7.5% compared with the base case. Full sensitivity analysis results for wage, travel, and caregiver parameters are presented in Multimedia Appendix 2.

We also tested the impact of the method used to calculate distance and travel costs within each CHSA by comparing the street distance method with the haversine method. We find that for 167 of 218 (76.6%) CHSAs, the haversine method was within 10 km of the street distance method. The remaining 51 of 218 (23.4%) had a difference greater than 10 km between the two methods; however, for some of the most remote regions, the haversine method underestimated the street distance by up to 137 km.

### Simulated Population

We applied our method to our simulated population to determine the total costs of ED, physician visits, and telehealth visits from the patient perspective over 1 year. Demographics and service use for the synthetic population are provided in Multimedia Appendix 1. Total costs are presented in Table 4, organized by service type, acuity, and health authority.

**Table 4:** Estimated patient-paid costs by service type and acuity for 1 year for a simulated population of 100,000.

Health Authority	Total ED^a^ CTAS^b^ I-III visit costs ($)	Total ED CTAS IV-V visit costs ($)	Total physician visit costs ($)	Total telehealth visit costs ($)
Fraser	2,292,674	982,692	8,508,221	2,261,474
Interior	1,005,335	431,293	3,987,006	972,891
Northern	424,195	187,685	1,985,431	368,011
Vancouver Coastal	1,350,332	561,400	3,758,996	1,487,933
Vancouver Island	934,499	387,732	3,009,011	1,020,796
Total	6,007,035	2,550,802	21,248,664	6,111,105

^a^ED: emergency department.

^b^CTAS: Canadian Triage Acuity Scale.

We see from this application of the method that substantial costs are likely paid by patients for both ED and physician visits. The simulation showed that ED and physician visits have a cost of just over $30,000,000 over 1 year per 100,000 people. While this total cost is not representative of current absolute costs, it illustrates the type of information that can be obtained by our costing method. Furthermore, we see that while physician visit costs are more than double those of ED visits, ED visits make up just 13.6% (42,573/312,573) of the total visits between the two. This suggests that ED visits are a relatively substantial cost driver despite being smaller in absolute number. In comparison, telehealth visits show considerably less expensive total costs than either ED or physician visits despite a comparable number of total visits to physicians. In addition, GHG emissions were calculated at varying proportions of hybrid care, which assumes both in-person and telehealth visits, the results of which are presented in Table 5.

**Table 5:** CO_2_-equivalent emissions for 1 year for a simulated population of 100,000.

			Estimated number of in-person visits		Total distance (km)		CO_2_-equivalent emissions (metric tons)
Physician visits			270,000		3,959,416		586.32
**Hybrid care**							
	25% in-person	67,500		989,854		146.58	
	50% in-person	135,000		1,979,708		293.16	
	75% in-person	202,500		2,969,562		439.74	
	ED^a^ visits	42,573		624,311		92.45	
	60% visits to the ED avoided by telehealth care	25,544		374,586.6		55.47	

^a^ED: emergency department.

When applying the method to a population, such as this simulated example, age group definitions can impact total cost output. Labor force participation may vary in complex ways with age or other factors, particularly when youth may start work and when individuals may retire [38,39]. To test the impact of age group definition calculated costs based on age group definitions of 0-19, 20-59, and ≥60 years and compared with the initial results. Total costs for each service type in Table 4 were reduced by 6%-8% with this age group definition.

## Discussion

### Principal Findings

Our study presents a novel method for estimating the patient-paid portion of costs attributable to receiving medical care with geography as our basis. The core calculation method and the categorization and the types of costs that are included can be applied to any set of parameters, whether they are based on routine data or customized values. Therefore, all parameters are flexible and can be swapped to those relevant to other jurisdictions, allowing the method to be generalizable to many different contexts. Furthermore, when applying the method to populations, labor force participation rates can be applied to change the age group definition or distribution to customized results for the population of interest. Our findings highlight the range and magnitude of cost variations depending on the type of care received, where patients live, and their age. This implies that economic evaluations of telehealth or in-person service from a patient’s perspective should consider a range of demographic characteristics of the relevant population to fully capture these costs. Our findings concur with previous research showing that a substantial portion of health care costs are passed off to patients when accessing care, particularly in rural areas [18]. Applying our method to a simulated population estimates that patients in British Columbia may collectively pay up to 30 million dollars per year to access health care services, primarily for medical travel.

### Comparison With Previous Work

Our findings add to the literature on patient-paid costs to access health care. These studies, however, are often tied to a specific cohort, using methods to determine travel and other out-of-pocket expenses that consider specific geographic data on patient locations and determine the distance traveled to either the nearest facility or the designated facility appropriate for the relevant treatment. Some methods also use patient location data and then calculate average distances to their facility [8]. Methods used by other researchers to calculate lost productivity—occasionally referred to as opportunity costs—account for total time and multiply by the relevant average wage [9,10,13]. Lost productivity costs using these methods have been estimated at $13.35 (2023 CAD equivalent) for a telehealth visit and $82.35 for an in-person visit [13]. An earlier study estimated the opportunity cost of an in-person visit to be $81.86 [9]. Our method found similar results, with lost productivity costs ranging from $42 to $166 for ED and physician visits. In addition, other studies do not report the per-visit cost directly but estimate cost savings of $124.10 for telehealth care [10]. Depending on the service being analyzed, calculations of patient lost productivity can be done over multiyear time horizons posttreatment and are thus geared toward providing insight on treatment outcomes rather than costs of visits directly. Travel distances, costs, and visit durations contributing to patient cost calculations are also often obtained from patient survey methods [5,7,40]. Survey results have shown that travel-related costs represent a substantial majority of the time spent and the costs that patients pay, with telehealth visits having considerably less cost [5,7,40]. Furthermore, studies of patient travel in Canada show that patients pay an average of $31 of the cost per person for travel to attend primary care appointments [14,41]. By including time costs by lost productivity and informal caregiving, we add the perspective of a comprehensive account of the total costs paid by patients that may be tailored depending on the scope of the economic evaluation. The World Health Organization’s methods of quantifying financial hardship currently refer to all costs attributed to out-of-pocket expenses at the time of receiving medical care; however, if there is an opportunity to save patients time during business hours by telehealth care provision, one may argue that accounting for lost productivity would be within the interest of patients, and applicable to providing more patient-centered care.

### Strengths and Limitations

Despite a motive for the deliberate embodiment of patient and family costs in evaluation, our method is primarily limited by our stated assumptions and the available evidence about what people pay to access health care. Specifically, our assumptions generalize anticipated differences in wages, labor force participation [38,39], the need for and availability of informal caregiving, fuel, vehicle, and meal costs. In addition, our analysis is limited to an account of the economic and environmental impacts of receiving medical care paid by patients, informal caregivers, and future generations. It does not consider the quality of care, which may result in further avoidable patient costs due to unnecessary services, nor does our method address questions related to the value of care. We rely on local travel maps and assumptions about the day-to-day travel considerations. In doing so, we likely underestimated the impact of seasonal variations, for example, during winter driving conditions or climate-related concerns such as road closures due to flooding or wildfires. The method, in its current form, does not account for constraints on the health care system, such as unanticipated ED closures that will cause patients to travel further. Furthermore, costs that are less easily monetized, such as those attributable to stress and emotional labor involved in emergencies and acute conditions, can create additional costs to patients that are less straightforward to quantify, especially with large distances between many patients and their usual health care centers. Therefore, this may result in the method underestimating the true costs to patients. The inclusion of the sensitivity analysis, while not addressing additional potential costs, is intended to mitigate the impact by showing how costs scale if more weight is given to particular parameters.

### Future Directions

This method is applicable to both research and policy contexts for economic evaluations of existing and newly proposed health care services. For example, studies calculating costs for rural health services, especially those with significant travel costs, could be augmented by our method in order to obtain a more comprehensive cost estimate and improve comparability between other studies and evaluations [40,42]. This may be particularly salient in travel reimbursement policies such as Canadian medical transport benefits [43]. Without a comprehensive and accurate calculation of patient costs, the level of burden placed on patients to access care can be unclear and likely underestimate these costs. While it is often indicated that access to care is a greater burden for those living in rural areas, without quantifiable cost information, certain geographic areas and populations may not receive sufficient resources and services. Using the method presented here will allow policymakers to more clearly understand the specific costs of accessing care for all patients in their jurisdiction and thus contribute to a more comprehensive evaluation to inform resource allocation.

### Conclusion

In this paper, we present a novel, generalizable method to estimate patient-paid costs and GHG emissions from accessing health care services. Through our geospatial costing method, we are able to show that patients pay substantial costs to access health services. Furthermore, in a simple simulation using historical data, we estimate that patients in British Columbia may be paying millions of dollars to access health care services. By including lost productivity, informal caregiving, and out-of-pocket expenses, the method is able to provide a more comprehensive and accurate calculation of patient-paid costs. Using this method to gain a better understanding of patient-paid costs will allow for more informed evaluations of new and existing services and aid in the comparison between potential alternatives.

## Appendix

Multimedia Appendix 1 Additional explanation of method, values of each parameter, and details of simulated population.

Multimedia Appendix 2 Full database of all unit costs obtained from our method. Full details of all sensitivity analyses completed.

## Abbreviations

CAA: Canadian Automobile Association
CHSA: community health service area
CIHI: Canadian Institute for Health Information
CTAS: Canadian Triage Acuity Scale
ED: emergency department
GHG: greenhouse gas
HA: health authority
NACRS: National Ambulatory Care Reporting System

## Data Availability

The datasets generated or analyzed during this study are available from the corresponding author on reasonable request.

## References

[1] Trenaman L, Kaal K, Laba T, Safari A, Aguiar M, Burch T, Beckett J, Munro S, Hudson M, Harrison M (2023). The financial burden of accessing care for people with scleroderma in Canada: a patient-oriented, cross-sectional survey. CMAJ Open.

[2] Rai KS, Mann U, Harasemiw O, Tangri N, Eng A, Patel P, Nayak JG (2023). A prospective evaluation of patient perspectives and financial considerations during prostate cancer treatment decision-making. Can Urol Assoc J.

[3] Cappitelli A, Wenzinger E, Langa O, Nuzzi L, Ganor O, Rogers-Vizena C, Ganske IM (2022). Cost and satisfaction implications of using telehealth for plagiocephaly. Plast Reconstr Surg Glob Open.

[4] Chan S, O'Riordan A, Appireddy R (2021). Exploring the determinants and experiences of senior stroke patients with virtual care. Can J Neurol Sci.

[5] Vallasciani S, Abdo B, Rauf Z, Anjum A, Ghulman S, Alghammas H, AlTaweel W (2019). Telehealth for the assessment of patients referred for pediatric urological care: a preliminary cost savings analysis and satisfaction survey. Telemed J E Health.

[6] Yabroff KR, Guy G, Ekwueme D, McNeel T, Rozjabek H, Dowling E, Li C, Virgo KS (2014). Annual patient time costs associated with medical care among cancer survivors in the United States. Med Care.

[7] Finkelstein JB, Cahill D, Young K, Humphrey K, Campbell J, Schumann C, Nelson CP, Gupta A, Estrada CR (2020). Telemedicine for pediatric urological postoperative care is safe, convenient and economical. J Urol.

[8] Geerdink TH, Geerdink N, van Dongen JM, Haverlag R, Goslings J, van Veen RN, Virtual Fracture Care Study Collaborative (2021). Cost-effectiveness of direct discharge from the emergency department of patients with simple stable injuries in the Netherlands. Trauma Surg Acute Care Open.

[9] Ray KN, Chari A, Engberg J, Bertolet M, Mehrotra A (2015). Opportunity costs of ambulatory medical care in the United States. Am J Manag Care.

[10] Head WT, Garcia D, Mukherjee R, Kahn S, Lesher A (2022). Virtual visits for outpatient burn care during the COVID-19 pandemic. J Burn Care Res.

[11] Jones G, Brennan V, Jacques R, Wood H, Dixon S, Radley S (2018). Evaluating the impact of a 'virtual clinic' on patient experience, personal and provider costs of care in urinary incontinence: a randomised controlled trial. PLoS One.

[12] White-Means S, Chollet D (1996). Opportunity wages and workforce adjustments: understanding the cost of in-home elder care. J Gerontol B Psychol Sci Soc Sci.

[13] Tselapedi-Sekeitto B, Rocha T, Sowerby L, Rotenberg B, Biadsee A (2023). Telemedicine as an environmental ally - the social, financial, and environmental impact of virtual care in the otolaryngology clinic. Am J Otolaryngol.

[14] Welk B, McArthur E, Zorzi A (2022). Association of virtual care expansion with environmental sustainability and reduced patient costs during the COVID-19 pandemic in Ontario, Canada. JAMA Netw Open.

[15] Ellis I, Cheek C, Jaffray L, Skinner T (2013). Making a case for telehealth: measuring the carbon cost of health-related travel. Rural Remote Health.

[16] Purohit A, Smith J, Hibble A (2021). Does telemedicine reduce the carbon footprint of healthcare? A systematic review. Future Healthc J.

[17] Miah S, Dunford C, Edison M, Eldred-Evans D, Gan C, Shah T, Lunn P, Winkler M, Ahmed HU, Gibbons N, Hrouda D (2019). A prospective clinical, cost and environmental analysis of a clinician-led virtual urology clinic. Ann R Coll Surg Engl.

[18] Croghan SM, Rohan P, Considine S, Salloum A, Smyth L, Ahmad I, Lynch TH, Manecksha RP (2021). Time, cost and carbon-efficiency: a silver lining of COVID era virtual urology clinics?. Ann R Coll Surg Engl.

[19] Paquette S, Lin J (2019). Outpatient telemedicine program in vascular surgery reduces patient travel time, cost, and environmental pollutant emissions. Ann Vasc Surg.

[20] Households with out-of-pocket payments greater than 40% of capacity to pay for health care (food, housing and utilities approach - developed by WHO/Europe).

[21] Mainer-Pearson G Geospatial-Patient-Costing-Method-Companion.

[22] Community Health Service Areas Boundaries - B.C. Health Region Master table - Version 2022.

[23] (2019). Community Health Service Areas - CHSA - Datasets - Data Catalogue. Government of British Columbia.

[24] CTAS introduction.

[25] Employee wages by industry, annual 2022. Statistics Canada.

[26] Retirement age by class of worker, annual. Statistics Canada.

[27] 2016 Census of Population: Age and sex release 2017. Statistics Canada.

[28] Hiring young people. BC Ministry of Labour.

[29] Differences in the characteristics of caregivers and caregiving arrangements of Canadians, 2018.

[30] Stall N (2019). We should care more about caregivers. CMAJ.

[31] CAA Driving Costs Calculator.

[32] Google Travel.

[33] Carbon Footprint Calculator.

[34] Minimum wage. British Columbia.

[35] NACRS emergency department visits and lengths of stay | CIHI.

[36] Irving G, Neves A, Dambha-Miller H, Oishi A, Tagashira H, Verho A, Holden J (2017). International variations in primary care physician consultation time: a systematic review of 67 countries. BMJ Open.

[37] Navigating Ethics Review. The University of British Columbia: Office of Research Ethics.

[38] Krol M, Hosseinnia N, Brouwer W, van Roijen LH (2023). Multiplier effects and compensation mechanisms for inclusion in health economic evaluation: a systematic review. Pharmacoeconomics.

[39] Brouwer W, Verbooy K, Hoefman R, van Exel Job (2023). Production losses due to absenteeism and presenteeism: the influence of compensation mechanisms and multiplier effects. Pharmacoeconomics.

[40] Kornelsen J, Khowaja A, Av-Gay G, Sullivan E, Parajulee A, Dunnebacke M, Egan D, Balas M, Williamson P (2021). The rural tax: comprehensive out-of-pocket costs associated with patient travel in British Columbia. BMC Health Serv Res.

[41] Recommendations for virtual care in primary care practices: a survey of patients across Ontario, Canada.

[42] Exploration of the costs of accessing health services: data from a longitudinal study of young people in transition from paediatric to adult services. BMC Health Services Research.

[43] Medical transportation benefits for First Nations and Inuit.

