# Supplementary material for: Estimating Patient and Family Costs and CO2 Emissions for Telehealth and In-Person Health Care Appointments in British Columbia, Canada: Geospatial Mixed Methods Study
Source: J Med Internet Res. 2025 Feb 19;27:e56766. doi: 10.2196/56766 (PMC11888102; doi:10.2196/56766)
Supplement: Multimedia Appendix 1 [file jmir_v27i1e56766_app1.docx]

## Supplementary Material

### Productivity changes

For physician visits, walk-in average wait time is found to be 58 minutes for BC, but as some physician appointments will be pre-scheduled, the estimate of 30 minutes is used for an average of all appointments. For physician visits, the summation of appointment time, wait time, and travel time is then considered the total time. For ED visits, the length of stay plus the travel time is considered the total time. All variables determining the total time commitment for either physician or emergency department visits can be found in Table 7.

Table 7. Parameters values & data sources.

| Data | Value | Source |
| --- | --- | --- |
| Travel/Car Costs | $0.48/km | CAA Car Costs Calculator [27] |
| Parking Costs | Location dependent | Municipal & Health Authority websites |
| Wage data | $30.54/hour | Statistics Canada |
| CHSA geographic data | - | Government of BC |
| Emergency Department List | - | HealthLink BC |
| Road network data | - | OpenStreetMap |
| Physician visit duration | 15.5 minutes | International physician wait-time study |
| Physician wait time | 30 minutes | MediMap |
| ED visit length of stay | 2.4-3.7 hours | CIHI |

### Out-of-Pocket Costs

For direct travel costs, the CAA car costs calculator was used with the assumption of a compact car being driven. This gives an average cost of $0.48 per kilometer, which is then multiplied by total distance travelled to get the total direct costs. For parking costs, a city from each health was chosen and the hourly rate (downtown for physician visit and hospital for ED) was then multiplied by the wait time plus appointment time (LOS for ED visits) to get total parking cost. Car maintenance costs are included in the $0.48/km value given by the CAA costs calculator.

### Data Usage Costs for Virtual Visits

For virtual visits, the primary source of costs for the direct out-of-pocket cost output is incurred via data usage to access the virtual service, either via connection to the internet or via telephone. For internet data usage, we estimated costs by determining the least expensive data plan in BC ($10/GB). We then consulted published material on average data usage per minute of a call across 10 wifi calling apps (529.46 kb/min). We then determined, using our average virtual visit duration and wait time parameters, that a virtual visit would incur $0.20 of data use. For virtual visits using telephones, we determine the least expensive landline phone plan in BC ($30/month) and consulted published sources on the average annual calling minutes used, which we calculated down to a per-month value (737.1 min/month per person). We then again used our virtual visit duration and wait time parameters to estimate a per visit cost of $1.51 for telephone data use. Finally, we averaged the per-visit cost values for telephone and internet data use (with 80% weight for telephone calls based on virtual visit usage patterns in BC) to obtain a final data usage cost value of $1.25 per visit for virtual visits.

### Simulated Population

**Table 8.** Simulated population demographics.

| Health Authority | Population | Age 0-14 | Age 15-64 | Age 65+ |
| --- | --- | --- | --- | --- |
| Northern | 6022 | 863 | 3935 | 1224 |
| Interior | 15920 | 2281 | 10403 | 3237 |
| Vancouver Coastal | 24348 | 3489 | 15909 | 4950 |
| Fraser | 37006 | 5303 | 24180 | 7523 |
| Vancouver Island | 16704 | 2394 | 10915 | 3396 |

**Table 9.** Simulated ED and Physician visits across British Columbia

| Health Authority | Physician Visits | ED CTAS I-III Visits | ED CTAS IV-V Visits | Virtual Visits |
| --- | --- | --- | --- | --- |
| Northern | 16259 | 1666 | 897 | 15055 |
| Interior | 42984 | 4405 | 2372 | 39800 |
| Vancouver Coastal | 99916 | 10240 | 5514 | 60870 |
| Fraser | 45101 | 4622 | 2489 | 92515 |
| Vancouver Island | 65740 | 6738 | 3628 | 41760 |
| Total | 270000 | 27672 | 14901 | 250000 |
